# Parental Ploidy Strongly Affects Offspring Fitness in Heteroploid Crosses among Three Cytotypes of Autopolyploid *Jacobaea carniolica* (Asteraceae)

**DOI:** 10.1371/journal.pone.0078959

**Published:** 2013-11-12

**Authors:** Michaela Sonnleitner, Birgit Weis, Ruth Flatscher, Pedro Escobar García, Jan Suda, Jana Krejčíková, Gerald M. Schneeweiss, Manuela Winkler, Peter Schönswetter, Karl Hülber

**Affiliations:** 1 Department of Systematic and Evolutionary Botany, University of Vienna, Vienna, Austria; 2 Institute of Botany, University of Innsbruck, Innsbruck, Austria; 3 Department of Botany, Faculty of Science, Charles University, Prague, Czech Republic; 4 Institute of Botany, Academy of Sciences of the Czech Republic, Průhonice, Czech Republic; 5 Department of Conservation Biology, Vegetation Ecology and Landscape Ecology, University of Vienna, Vienna, Austria; 6 Vienna Institute for Nature Conservation & Analyses, Vienna, Austria; University of Minnesota, United States of America

## Abstract

Reproductive interactions among cytotypes in their contact zones determine whether these cytotypes can co-exist and form stable contact zones or not. In autopolyploids, heteroploid cross-compatibilities might depend on parental ploidy, but tests of this hypothesis in autopolyploid systems with more than two ploidies are lacking. Here, we study *Jacobaea carniolica*, which comprises diploid, tetraploid, and hexaploid individuals regularly forming contact zones. Seeds obtained from *in situ* cross-pollinations within and among cytotypes were subjected to DNA flow cytometry and greenhouse germination experiments. Hybrid fitness and parental effects on hybrid fitness were tested with regression models comparing fitness parameters of early life stages. Irrespective of the direction of crosses, seed viability and seedling survival in diploid-polyploid crosses were substantially lower than in tetraploid-hexaploid crosses. In contrast, seedling growth traits indicated neither transgressive character expression nor any selection against hybrid offspring. Congruent with a model of genome dosage effects, these traits differed between reciprocal crosses, especially of diploids and tetraploids, where trait values resembled those of the maternal parent. The strong effect of parental ploidy on offspring fitness in heteroploid crosses may cause contact zones involving exclusively polyploid cytotypes to be less stable over longer terms than those involving diploids and polyploids.

## Introduction

Polyploidisation, i.e. the multiplication of the whole genome, is among the most important evolutionary mechanisms in plants, accounting for 15 and 31% of speciation events in angiosperms and ferns, respectively [1]. Even though genome multiplication per se is widely accepted as a possible mechanism for instantaneous speciation [2], it usually does not confer complete reproductive isolation of the polyploid from its lower-ploid ancestors. Therefore, other adaptive or neutral processes are necessary to enable coexistence of both cytotypes [3]. Much research has focussed on the early phases of polyploid establishment, such as the frequency-dependent mating disadvantage of newly emerged polyploids (minority cytotype exclusion principle; [4], [5]), the role of fitness advantages [6], [7] or niche differentiation [8], [9], and on cytotype interfertility among established polyploids and/or their ancestors [10]–[17].

Minority cytotype exclusion and fitness differences among cytotypes are not only significant for neopolyploid establishment, but are assumed to be equally important in secondary contact zones [11]. Such areas where cytotypes regain contact after a period of divergence in allopatry are common [18] and frequently arose in the course of (postglacial) range expansions [19]–[21]. They are evolutionary melting pots enhancing local diversity [22] and may ultimately even promote speciation [23].

The extent of reproductive isolation among cytotypes determines the long-term integrity of lineages in any contact zone [24]. Mechanisms of reproductive isolation include ecological niche differentiation [25]–[27], flowering time divergence [26], pollinator preferences [28], [29], pollen competition and genetic/genomic incompatibilities preventing zygote and/or endosperm formation [30]–[32], or negative effects on vitality and fertility of hybrid offspring from seed formation to fertility in the F_1_ or even later generations [11]. Frequently, several mechanisms act in concert allowing the coexistence of closely related lineages [33], [34]. Reproductive isolation mechanisms between a polyploid and its lower-ploid ancestor(s) have been studied both in allopolyploids [35], [36] and in autopolyploids [14], [27], [33], [37], [38]. As these systems often comprise only a single polyploid cytotype (tetraploid or hexaploid, but see: [15], [39]), little is known whether reproductive isolation mechanisms and/or their strength differ in dependency of the ploidy.

Postzygotic isolation may be conferred by genome imbalance in the endosperm [40] frequently leading to seed abortion in heteroploid crosses [41], [42]. A ratio of two maternal (2 m) and one paternal (1p) genomes in the endosperm is critical for seed maturation in many species [5], [43]–[45], whereas the embryo itself is often more resistant to differences in parental ploidies [46]. Mechanisms underlying this dosage effect include cytoplasmatic effects and/or genomic imprinting [42], the latter referring to the epigenetic phenomenon of functional non-equivalence of maternal and paternal genes despite their identical DNA sequence [40]. Since the magnitude of deviation from a balanced endosperm depends on parental ploidy (e.g. two-fold versus three-fold in diploid-tetraploid versus diploid-hexaploid crosses), the extent of offspring fitness reduction might also be expected to vary between different heteroploid crosses, but this hypothesis has been insufficiently tested so far. Furthermore, the direction of the deviation from a balanced endosperm can have complementary phenotypic consequences [42], [47]. Specifically, an excess of maternal genomes induces accelerated endosperm cellularisation and delayed mitosis, resulting in small embryos, whereas paternal excess leads to delayed cellularisation and accelerated mitosis in the endosperm producing large embryos [46]. This will affect seed size and thus offspring fitness and may even cause seed abortion, resulting in cross-incompatibility termed triploid [5] or, more generally, inter-ploidy block [39]. Consequently, the fitness of hybrid offspring might also depend on the direction of heteroploid crosses (e.g., refs. [14], [16]).

Comprehensive knowledge of heteroploid cross-compatibility including hybrid fitness is, hence, important to understand the dynamics of contact zones with respect to, for instance, the spatial configuration of contact zones, competition avoidance of cytotypes or gene-flow between cytotypes potentially leading to the formation of new hybrids or the elimination of one cytotype in natural habitats [15], [24], [48]. Our study presents the results of reciprocal *in situ* cross-pollinations in the alpine plant *Jacobaea carniolica* (Asteraceae). This group comprises three main cytotypes (diploids, tetraploids, hexaploids), which form contact zones in every conceivable combination [49]. The occurrence of pentaploids in tetraploid-hexaploid contact zones and the lack of tetraploids in diploid-hexaploid contact zones [48], [49] suggest ploidy-dependent differences in cross-compatibility, but these inferences might be biased as early life stages are hard to find in nature and, therefore, have not been considered in the previous investigations. Here, we test for differences in cross-compatibility in all combinations of heteroploid crosses evaluating four fitness components of early life stages (encompassing hybrid formation and seedling performance). Specifically, we aim to answer the following questions: (1) What is the extent of cross-compatibility between cytotypes and which fitness components are most strongly affected by reproductive incompatibility? (2) Is there any evidence for selection against hybrids? Are the characters of hybrids intermediate, parental-like or transgressive? (3) Do the magnitude and/or the direction of deviation from a balanced endosperm affect seed formation and/or seedling fitness?

## Materials and Methods

### Ethics Statement

Field studies were carried out on private land with oral permissions of the owners (Schwarzenberg’sche Forstverwaltung and Jacques Lemans GmbH, St. Veit an der Glan). Field studies did not involve endangered or protected species.

### Study Species

*Jacobaea carniolica* (Willd.) Schrank (syn. *Senecio carniolicus* Willd.) is a herbaceous perennial common on acidic bedrock in the alpine to subnival belt of the Eastern Alps and the Carpathians. The polyploid complex comprises mainly diploids, tetraploids and hexaploids in the Eastern Alps and only hexaploids in the Carpathians [49], [50]. In contrast to the majority of polyploid complexes, *J. carniolica* does not form a single contact zone containing otherwise geographically well separated cytotypes (e.g., refs. [51]–[54]), but occurs in various combinations of cytotypes throughout major parts of the Eastern Alps [49], [50]. Of 100 investigated sample sites, diploids and hexaploids, tetraploids and hexaploids, and diploids and tetraploids co-occurred at 28, five and three sites, respectively, and all three cytotypes co-occurred in eight sample sites. Molecular genetic evidence suggests that the polyploid cytotypes are autopolyploid derivatives of a diploid lineage distributed in the easternmost Alps ([55], M. Winkler et al., unpublished data). Strong genetic divergence between the ancestral eastern diploid lineage and its polyploid derivatives renders ongoing polytopic origin of the polyploids unlikely (M. Winkler et al., unpublished data), which is in line with a consistent morphological differentiation of diploids and polyploids [56]. Despite substantial habitat segregation [48], individuals of different cytotypes commonly occur in close spatial proximity (less than one meter; [49]), potentially enabling *in situ* heteroploid pollination. In addition, flowering times overlap strongly (M. Sonnleitner, pers. obs.) enabling artificial crossing throughout the flowering time of all three cytotypes and generalistic behaviour of alpine pollinators [57] precludes a strong pre-pollination isolation.

### Artificial Crosses

Reciprocal *in situ* cross-pollinations were conducted during summer of 2009 east of Turracher Höhe (Gurktaler Alps, Austria, N 46.91 E 13.92 at *c.* 2250 m a.s.l.), where pairwise contact zones of the three main cytotypes occur. Using individuals from a single population avoids that effects of ploidy, the focus of this study, are confounded with those of geographic differentiation due to, for instance, adaption to different ecoclimatic conditions. Each cytotype was either outcrossed with itself (i.e. homoploid crosses) or with other cytotypes (heteroploid crosses), or self-pollinated to test for self-incompatibility (SI; Table 1). The ploidy of all crossing partners was determined via flow cytometry (FCM) of silica-dried leaf material following Sonnleitner et al. [49]. Each inflorescence serving either as pollen receptor or as pollen donor was bagged before anthesis using a small-meshed tissue to prevent uncontrolled pollen transfer. As the floret morphology in *Jacobaea* precluded emasculation, self-pollination could not be avoided, but was found to be low (see Results); as cytotypes did not differ in their selfing levels, the quantitative effects of selfing on the measured fitness parameters (via, for instance pollen competition or interference) are expected to be similar for all cytotypes. Hand-pollinations were done by rubbing anthetic flower heads of mother plants and pollen donor plants [14], [58], or using a brush in case of selfing. Inflorescences were kept in bags for seven to eight weeks and harvested at seed maturity. After harvest, cypselas (termed “seeds” in the following for simplicity) were stored at 4°C. Seeds were visually classified as viable (firm and plump pericarp) and non-viable (empty pericarp). Prior dissections of soaked seeds were used to assess the accuracy of this classification. To discriminate hybridogenic and selfed seeds, determination of the ploidy is essential. As the ploidy status of tiny seeds currently cannot be determined in a non-destructive way, viable seeds of each mother plant were split into two groups of equal size designated for germination and for cytotype determination via FCM, respectively.

**Table 1:** *In situ* cross-pollinations (left) among diploid, tetraploid and hexaploid individuals of *Jacobaea carniolica* and consecutive germination of a subset of the seed yield of heteroploid crosses (right) in the climate chamber.

**Treatment** 1	**no. mother plants**	**no. florets (ovules)**	**seed viability %**	**no. of seeds cytotyped**	**% of cytotyped seeds by ploidy** **(hybrids given in bold)**						**no. of exposed seeds**	**surviving seedlings** **no./%**	**% of surviving seedlings according to estimated ploidies (hybrids given in bold)**						
					**2** ***x***	**3** ***x***	**4** ***x***	**5** ***x***	**6** ***x***	**7** ***x*** **/8** ***x***			**2** ***x***	**3** ***x***	**4** ***x***	**5** ***x***	**6** ***x***	**7** ***x*** **/8** ***x***	**failed**
2*x*4*x*	39	3955	11.1	121	21.5	**69.4**	9.1	–	–	–	195	58/29.7	48.3	**41.4**	3.4	1.7	–	–	5.2
4*x*2*x*	37	5878	13.8	123	–	**69.1**	30.9	–	–	–	212	63/29.7	–	**52.4**	30.1	3.2	–	–	14.3
2*x*6*x*	40	4290	2.9	43	72.1	–	**23.3**	4.6	–	–	56	16/28.6	81.2	–	–	6.3	–	–	12.5
6*x*2*x*	36	5692	7.6	57	–	–	**31.6**	28.1	40.3	–	102	40/39.2	–	–	**5.0**	27.5	57.5	–	10.0
4*x*6*x*	34	5624	39.4	154	–	–	3.9	**95.4**	–	0.7	286	181/63.3	–	–	1.1	**90.1**	1.1	–	7.7
6*x*4*x*	39	6107	38.5	186	–	–	–	**92.5**	5.9	1.6	365	192/52.6	–	–	–	**88.5**	3.7	1.0	6.8
2*x*2*x*	40	3858	52.2	103	100	–	–	–	–	–	405	149/36.8							
4*x*4*x*	36	5614	50.5	122	–	–	100	–	–	–	334	100/29.9							
6*x*6*x*	38	5822	32.6	154	–	–	–	–	100	–	354	207/58.5							
2*x*SI	31	3095	4.6								92	39/42.4							
4*x*SI	29	4268	7.7								130	35/26.9							
6*x*SI	28	4966	1.3								54	29/53.7							

1 maternal parent is given first; SI, selfing treatments.

### Flow Cytometry of Seeds (FCM)

Ploidy of dry seeds was estimated using 4′,6-diamidino-2-phenylindole (DAPI) FCM. Basically, we adopted the methodology described previously [50] with the following modifications: (i) the samples were run on a flow cytometer *c.* 30 s after the addition of the staining solution (longer incubation period resulted in a lower histogram quality), (ii) *Pisum sativum* ‘Ctirad’ [59] was used as the sole internal reference standard, (iii) fluorescence intensity of 3000 particles was recorded, (iv) manual gating of the embryo and the endosperm peaks was occasionally applied (particularly in analyses with a relatively low yield of intact nuclei). Two seeds were usually analysed together. The flow histograms mostly consisted of three peaks: (i) nuclei of the internal reference standard (usually having the lowest coefficient of variation, CV), (ii) embryo nuclei and (iii) endosperm nuclei. The peak corresponding to endosperm nuclei was usually distinctly smaller than that of embryo nuclei; in rare cases it was completely lacking. The quality threshold (maximum acceptable CV values) for the embryo peak was relaxed to 8%.

### Germination Experiment

If available, ten potentially viable seeds per mother plant, resulting in a total of 2585 seeds, were germinated on moist filter paper in petri dishes exposed in a climate chamber (Heraeus Vötsch NPS1500 S-CTC) using settings closely resembling *in situ* conditions: day/night 14/10 hrs, temperature 15/5°C; temperature adaptation within 1 hr, 90% constant relative humidity (rH), approximately 370 µmol photons m^−2 ^s^−1^ light (Osram powerstar HQ I-R 250W/NDL neon lights) during day time. Petri dishes were controlled, watered and repositioned daily to avoid edge effects. Seedlings with fully expanded cotyledons were transplanted into multipot plates, each pot containing *c.* 150 ml of substrate (coco fibre, sand, Osmocote™ depot-fertiliser, proportions approximately 90∶9∶1) and further cultivated in acclimatisation chambers with 60% rH, *c.* 22°C temperature, automatic watering and a day-length of 14 hrs. Every fourth day the vitality of seedlings was controlled and the appearance of primary leaves was recorded. At the end of the experiment (i.e. approximately seven weeks after exposure), for each rosette the largest diameter and the largest perpendicular to it, the number of leaves and the maximum leaf length were measured and one of the cotyledons was sampled for ploidy determination via FCM (initial analyses found no incidence of endopolyploidy in cotyledons: data not shown).

### Statistical Analyses

The effects of ploidy of crossing partners (i.e. treatments) on the reproductive success were analysed by means of four fitness components of F_1_-individuals: (1) *Seed viability* represents the state of seeds classified as viable/non-viable; (2) *Survival of seedlings* represents the vitality of individuals exposed in the climate chamber at the end of the experiment coded as alive/dead; (3) The *time from exposure of seeds to the appearance of the primary leaf* given in days; and (4) the *size of seedlings* described by the first axis (72.3% of total variation) of a principal component analysis (PCA) using the number of leaves, the length of the longest leaf and the two rosette diameters measured at the end of the experiment. Offspring of heteroploid crosses may show intermediate ploidy due to hybridisation, the maternal ploidy due to selfing, or increased ploidy due to the involvement of unreduced gametes [12], [32]. Whereas seed viability comprised the overall seed yield only individuals with intermediate ploidy (referred to as hybrid seedlings in the following) were used to estimate the other fitness components of seedlings.

We analysed the effects of parental ploidy on the four fitness components described above by means of linear mixed-effects models (LMM, in case of size of hybrid seedlings) or generalised linear mixed-effects models (GLMM, in case of the three other fitness components). Models relate one fitness component as response to the treatment as fixed-effect predictor. Since for these analyses data of more than one seed or seedling from each pollinated individual were used, we accounted for the potential effects of this dependence by using mother plant as a grouping variable and allowing for a random intercept for each group. For the binomially distributed data of seed viability and survival of hybrid seedlings we used the canonical logit link-function. To model the time to emergence of the primary leaf and the size of hybrid seedlings, Poisson (log-link) and Gaussian (identity-link) distributions of errors, were assumed, respectively. Parameters were estimated by restricted maximum likelihood in case of the LMM and based on the Laplacian approximation in case of the logistic and Poisson GLMM. For the Gaussian models the degrees of freedom were calculated as n_observations_ – n_fixed effects_ – n_random effects_ + n_random terms_.

We tested the expectation that in case of no selection against hybrid offspring the fitness of F_1_-individuals of heteroploid crosses, measured by seed viability and seedling survival, is intermediate to that of the parental cytotypes or at least not lower than the worse performing parental cytotype. Thus, by using Helmert contrasts, each heteroploid crossing treatment was compared to the pooled data of homoploid crosses of the parental cytotypes (in the following referred to as parental treatments), e.g. 2*x*4*x* (maternal parent given first) was compared to pooled 2*x*2*x* and 4*x*4*x*. In case of hybrid fitness not intermediate between parental treatments we evaluated if hybrid fitness differed from that of the worse performing parental cytotype. Furthermore, we tested whether phenotypic fitness traits, time to primary leaf and seedling size, are intermediate, parental-like or transgressive using the same approach as described above, but in case of non-intermediacy fitness parameters were compared with the more similar parental treatment. Finally, to detect maternal or paternal effects on hybrid fitness, treatments comprising the same parental cytotypes but with reversed direction of crosses were tested, e.g. 2*x*4*x* was compared to 4*x*2*x*.

As the ploidy of seeds that failed to germinate and of dead or dying seedlings usually cannot be determined, the ploidy of seedlings is only known if they survived until the end of the experiment when leaf/cotyledon material was collected for FCM. Thus, it is unknown whether an individual that has died during the experiment originated from selfing or hybridisation, which makes a straightforward estimation of the survival of hybrid seedlings impossible. Therefore, we assumed an equal percentage of hybrid seeds in the germination experiment and among the seeds analysed with FCM. A subset of the size nHyb_dead_ of dead individuals was randomly selected and defined as hybrids in the survival models following the formula
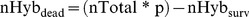
(1)where nTotal represents the number of seeds exposed in the germination experiment, p is the proportion (ranging from 0 to 1) of hybrid seeds in the FCM analysis, and nHyb_surv_ and nHyb_dead_ are the numbers of surviving and dead hybrid seeds in the germination experiment, respectively. Coefficients and z-values given for survival models represent means of 100 repetitions of this random association of dead progeny to mother plants and subsequent model fitting. P-values of models of seedling survival were calculated using the mean of z-values.

All statistical analyses were computed in R 2.13.0 [60]. LMM and GLMM were fitted using the glmer-function implemented in the lme4-package [61].

## Results

**Figure 1:**
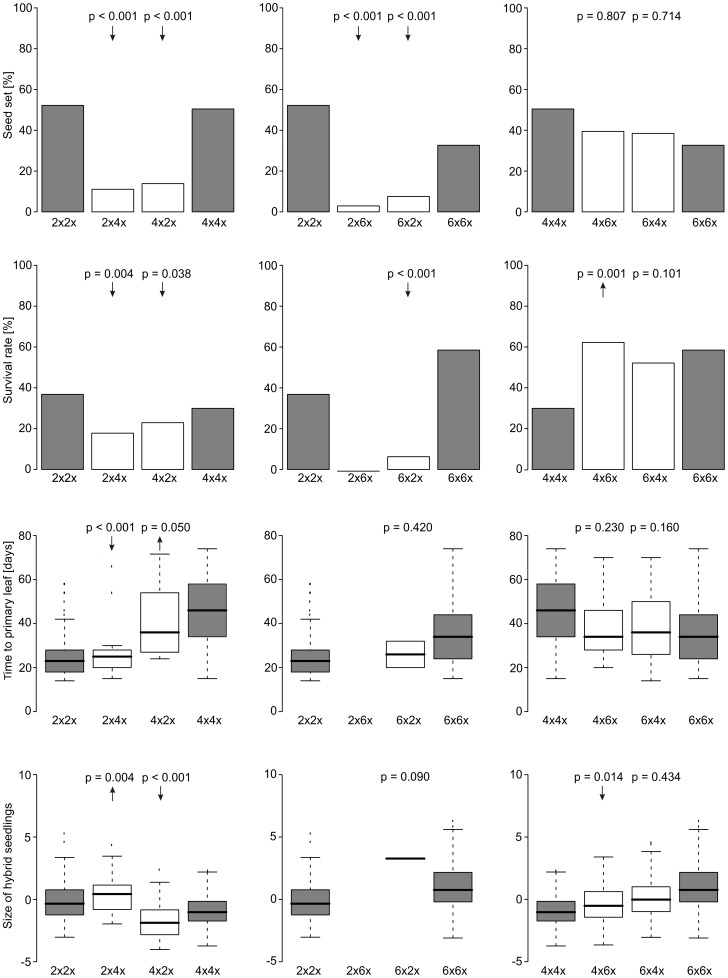
Fitness of progeny. Comparison of the fitness of progeny derived from homoploid (grey bars) or heteroploid crosses (white bars) of the three main cytotypes of *Jacobaea carniolica*. Seed set and survival rate represent the proportion of viable seeds and the proportion of seedlings alive at the end of the experiment, respectively. For heteroploid crosses only hybrids (ploidy intermediate to parental ploidies) were considered, except for seed set. P-values derived from (generalised) linear mixed-effects models indicate significance of deviations of heteroploid progeny (e.g. 2*x*4*x*, 4*x*2*x*; maternal parent is given first) from the intermediate value of parental homoploid crosses (e.g. 2*x*2*x*, 4*x*4*x*); arrows indicate the direction of significant deviations.

A total of 427 plants were pollinated in the crossing experiments (see Table 1 for an overview of the crossing and germination experiment). Each diploid, tetraploid and hexaploid mother plant had on average 101, 157 and 160 florets, respectively. Seed viability was strongly affected by the ploidy of crossing partners (Table 1). Diploid-polyploid crosses showed significantly lower seed viability than both the pooled (Figure 1, Table 2) and the worse performing parental treatment (Table 3). In contrast, seed viability of tetraploid-hexaploid crosses was intermediate between the parental treatments (Table 2). The direction of crosses had no significant effect on the seed viability for any combination of cytotypes (Table 4). Seed viability in all SI-treatments was low (<10%), indicating a low selfing capability of all cytotypes (Table 1).

**Table 2:** Test for intermediacy of fitness components of heteroploid crosses of *Jacobaea carniolica*.

Comparison of treatments^1,2^	no. total^3^	no. hybrids^4^	coefficient ± SE	z- or t- value	p-value^5^
**Seed viability**					
(2*x*2*x*,4*x*4*x*) ↔ 2*x*4*x*	13427/115	–	−0.82±0.08	−9.97	**<0.001**
(2*x*2*x*,4*x*4*x*) ↔ 4*x*2*x*	15350/113	–	−0.83±0.09	−9.30	**<0.001**
(2*x*2*x*,6*x*6*x*) ↔ 2*x*6*x*	13970/118	–	−1.22±0.10	−12.74	**<0.001**
(2*x*2*x*,6*x*6*x*) ↔ 6*x*2*x*	15372/114	–	−1.10±0.11	−9.75	**<0.001**
(4*x*4*x*,6*x*6*x*) ↔ 4*x*6*x*	17060/108	–	−0.03±0.11	−0.24	0.807
(4*x*4*x*,6*x*6*x*) ↔ 6*x*4*x*	17543/113	–	−0.03±0.09	−0.37	0.714
**Survival of hybrid seedlings**					
(2*x*2*x*,4*x*4*x*) ↔ 2*x*4*x*	874	135	−0.29±0.10	−2.91	**0.004**
(2*x*2*x*,4*x*4*x*) ↔ 4*x*2*x*	883	147	−0.18±0.09	−2.08	**0.038**
(2*x*2*x*,6*x*6*x*) ↔ 2*x*6*x*	–	–	–	–	–
(2*x*2*x*,6*x*6*x*) ↔ 6*x*2*x*	791	32	−0.95±0.29	−3.30	**<0.001**
(4*x*4*x*,6*x*6*x*) ↔ 4*x*6*x*	950	273	0.28±0.09	3.12	**0.001**
(4*x*4*x*,6*x*6*x*) ↔ 6*x*4*x*	1014	338	0.13±0.08	1.64	0.101
**Time to primary leaf in hybrid seedlings**					
(2*x*2*x*,4*x*4*x*) ↔ 2*x*4*x*	326/86	26/16	−0.10±0.02	−4.10	**<0.001**
(2*x*2*x*,4*x*4*x*) ↔ 4*x*2*x*	331/87	31/17	0.05±0.02	1.96	**0.050**
(2*x*2*x*,6*x*6*x*) ↔ 2*x*6*x*	–	–	–	–	–
(2*x*2*x*,6*x*6*x*) ↔ 6*x*2*x*	416/75	2/2	−0.05±0.07	−0.81	0.420
(4*x*4*x*,6*x*6*x*) ↔ 4*x*6*x*	486/95	160/30	−0.02±0.01	−1.20	0.230
(4*x*4*x*,6*x*6*x*) ↔ 6*x*4*x*	495/101	169/36	−0.02±0.02	−1.40	0.160
**Size of hybrid seedlings**					
(2*x*2*x*,4*x*4*x*) ↔ 2*x*4*x*	260/83	23/15	0.33±0.11	2.91	**0.004**
(2*x*2*x*,4*x*4*x*) ↔ 4*x*2*x*	268/87	31/19	−0.38±0.10	−3.75	**<0.001**
(2*x*2*x*,6*x*6*x*) ↔ 2*x*6*x*	–	–	–	–	–
(2*x*2*x*,6*x*6*x*) ↔ 6*x*2*x*	346/71	1/1	0.96±0.57	1.70	0.090
(4*x*4*x*,6*x*6*x*) ↔ 4*x*6*x*	456/94	158/30	−0.18±0.07	−2.46	**0.014**
(4*x*4*x*,6*x*6*x*) ↔ 6*x*4*x*	467/100	169/36	0.05±0.07	0.78	0.434

1 maternal parent is given first;

2 pooled data of the homoploid crosses of the parental cytotypes were compared to heteroploid crosses by means of (generalised) linear mixed-effects models;

3 number of observations (i.e. number of pollinated florets of all treatments in a comparison) and number of groups (i.e. number of pollinated plants) used in the models;

4 number of flow-cytometrically verified hybridogenic seedlings and number of pollinated plants from which these hybrids originate (see text for details);

5 p-values ≤0.05 are given in bold.

**Table 3:** Test for parental-like fitness components of heteroploid crosses of *Jacobaea carniolica*.

Comparison of treatments^1^	no. total^2^	coefficient ± SE	z- or t- value		p-value^3^
**Seed viability**					
2*x*4*x* ↔ 4*x*4*x*	9569/75	−2.37±0.31	−7.69		**<0.001**
4*x*2*x* ↔ 4*x*4*x*	11492/73	−2.41±0.34	−7.00		**<0.001**
2*x*6*x* ↔ 6*x*6*x*	10112/78	−3.14±0.37	−8.53		**<0.001**
6*x*2*x* ↔ 6*x*6*x*	11514/74	−2.79±0.46	−6.12		**<0.001**
**Survival of hybrid seedlings**					
2*x*4*x* ↔ 4*x*4*x*	469	−0.70±0.34	−2.09		**0.037**
4*x*2*x* ↔ 4*x*4*x*	478	−0.38±0.28	−1.35		0.177
2*x*6*x* ↔ 2*x*2*x*	–	–	–		–
6*x*2*x* ↔ 2*x*2*x*	437	−2.24±0.82	−2.72		**0.007**
4*x*6*x* ↔ 4*x*4*x*	596	1.56±0.30	5.22		**<0.001**
4*x*6*x* ↔ 6*x*6*x*	616	0.15±0.33	0.44		0.659
**Time to primary leaf in hybrid seedlings**					
2*x*4*x* ↔ 2*x*2*x*	220/55	−0.02±0.07	0.32		0.748
4*x*2*x* ↔ 4*x*4*x*	137/48	−0.13±0.08	−1.62		0.106
**Size of hybrid seedlings**					
2*x*4*x* ↔ 2*x*2*x*	165/52	−0.59±0.35	1.69	0.094	
4*x*2*x* ↔ 4*x*4*x*	126/50	−0.78±0.33	−2.37	**0.020**	
4*x*6*x* ↔ 4*x*4*x*	253/61	0.39±0.24	1.59	0.114	

1 maternal parent is given first; calculated only for significant comparisons in Table 2;

2 number of progeny and number of pollinated plants used in the (generalised) linear mixed-effects models;

3 p-values <0.05 are given in bold.

**Table 4:** Influence of the direction of heteroploid cross-pollinations in *Jacobaea carniolica* on the fitness of the progeny.

Comparison of treatments^1^	no.^2^	coefficient ± SE	z- or t- value	p-value^3^
**Seed viability**				
2*x*4*x* ↔ 4*x*2*x*	9833/76	−0.02±0.33	−0.08	0.940
2*x*6*x* ↔ 6*x*2*x*	9982/76	0.45±0.43	1.04	0.299
4*x*6*x* ↔ 6*x*4*x*	11731/73	−0.02±0.34	−0.05	0.963
**Survival of hybrid seedlings**				
2*x*4*x* ↔ 4*x*2*x*	279	−0.32±0.34	−0.93	0.352
2*x*6*x* ↔ 6*x*2*x*	–	–	–	–
4*x*6*x* ↔ 6*x*4*x*	588	0.44±0.33	1.31	0.190
**Time to primary leaf in hybrid seedlings**				
2*x*4*x* ↔ 4*x*2*x*	57/33	0.43±0.10	4.31	**<0.001**
2*x*6*x* ↔ 6*x*2*x*	–	–	–	–
4*x*6*x* ↔ 6*x*4*x*	329/66	−0.01±0.06	−0.23	0.816
**Size of hybrid seedlings**				
2*x*4*x* ↔ 4*x*2*x*	54/34	−2.20±0.50	−4.41	**<0.001**
2*x*6*x* ↔ 6*x*2*x*	–	–	–	–
4*x*6*x* ↔ 6*x*4*x*	327/66	0.68±0.21	3.17	**0.002**

1 maternal parent is given first;

2 number of seeds/hybridogenic seedlings and pollinated plants used in the (generalised) linear mixed-effects models;

3 p-values <0.05 are given in bold.

The proportion of hybrids (seeds and seedlings with intermediate ploidy) of total progeny in heteroploid crosses was strongly affected by their parental cytotypes (Table 1). Tetraploid-hexaploid, diploid-tetraploid and diploid-hexaploid crosses yielded the highest, intermediate and lowest numbers of hybrids, respectively. The proportion of seeds and seedlings derived from selfing (showing the maternal ploidy) was high in all diploid-polyploid and low in tetraploid-hexaploid crosses. Progeny with irregular ploidy due to the involvement of unreduced gametes was mainly produced in diploid-hexaploid crosses, particularly with maternal hexaploids (6*x*2*x* with a siring success of unreduced diploid pollen of 28.1%; Table 1), but rare in tetraploid-hexaploid crosses (siring success of unreduced gametes of less than 2%; Table 1). Homoploid treatments yielded only seeds of the parental ploidy. In diploid-tetraploid crosses it is not possible to distinguish with FCM triploids of hybridogenic origin (2*x*4*x*) from triploids produced via selfing of diploids with unreduced pollen. Likewise, tetraploids stemming from selfing cannot be discriminated from hybridogenic tetraploids involving unreduced diploid pollen (4*x*2*x* treatment; Table 1). The overall reproductive success, i.e. the proportions of hybrid seedlings alive at the end of the experiment related to the total number of ovules/florets pollinated, was 1.37/2.18% in 2*x*4*x*/4*x*2*x*, <0.01/0.15% for 2*x*6*x*/6*x*2*x* and 23.37/18.53% for 4*x*6*x*/6*x*4*x* crosses. Nuclear DNA amounts of offspring corresponded well with those of parental plants of the same ploidy level.

Survival of diploid-polyploid hybrid seedlings was significantly lower than the average survival of seedlings emerging from parental treatments (Figure 1, Table 2). This is particularly evident in 2*x*6*x* crosses, where not a single tetraploid hybrid survived until the end of the experiment. Hybrids with a diploid parent most often performed inferior to the worse performing parent (only 4*x*2*x* did not significantly differ from 4*x*4*x*; Table 3). Pentaploids originating from tetraploid-hexaploid crosses either showed intermediate (6*x*4*x*) or parental-like (4*x*6*x* resembling 6*x*6*x*) survival compared to the parental treatments. Again, the direction of crosses did not influence the survival of hybrid offspring (Table 4).

The time to develop the primary leaf in pentaploid seedlings was intermediate to that of parental treatments (Figure 1, Table 2) with no effect of crossing direction (Table 4). In contrast, triploid seedlings developed significantly faster (2*x*4*x*) or slower (4*x*2*x*) than the expected intermediate level (Table 2). However, hybrids between diploids and tetraploids did not differ significantly from the respective maternal treatment (Table 3). Only two out of 32 surviving hybrid seedlings of the 6*x*2*x* and none of the 2*x*6*x* treatment developed a primary leaf rendering statistical tests impossible.

The size of hybrid seedlings was larger (2*x*4*x*) or smaller (4*x*2*x*, 4*x*6*x*) than the average of the parental treatments (Figure 1, Table 2). The size of triploid hybrids thus reflects their developmental speed, since faster-growing seedlings also reach larger size. The 4*x*2*x* seedlings were significantly smaller than plants derived from both parental treatments (Table 3), representing the only case of transgressive character expression. The direction of crosses significantly influenced the size of triploid and pentaploid hybrid seedlings, which was larger when the lower ploidy acted as maternal parent (Table 4).

## Discussion

### Parental Cytotypes Determine Success of Heteroploid Crosses

Reproductive interactions of cytotypes of *Jacobaea carniolica* in secondary contact zones containing diploids and their autopolyploid derivatives (i.e. tetraploids and hexaploids) differed strongly depending on the cytotypes involved. Seed viability and survival of seedlings resulting from crosses of diploids with polyploids were significantly reduced as compared to the offspring of homoploid parental treatments, indicating strong selection against hybrid offspring. In particular, cross-pollinations between diploids and hexaploids, i.e. the cytotypes co-occurring most frequently throughout the Eastern Alps [49], [50], often within a few decimetres [48], produced only a few seeds with very low or even no germination success and seedling survival (Figure 1, Table 2). Reproductive isolation between diploids and tetraploids was slightly weaker than for diploid-hexaploid crosses with respect to seed viability and seedling survival (Figure 1). In both diploid-tetraploid and diploid-hexaploid crosses, the few surviving seedlings showed no evidence for phenotypic inferiority compared to homoploid seedlings, with the possible exception of seedling size in 4*x*2*x* crosses (Figure 1, Table 3). In contrast to heteroploid crosses involving diploids, reproductive success between polyploid cytotypes was high; seed viability and/or seedling performance parameters were either intermediate between parental treatments or equal to the better performing parental treatment (Figure 1, Table 2). We found no evidence for transgressive character expression in favour of hybrids, at least in early life stages, since hybrid fitness in none of the treatments significantly exceeded the fitness of the better performing parental cytotypes (Table 3).

Reproductive success of heteroploid crosses between diploids and (sexual) autopolyploid tetraploids varies strongly among taxonomic groups ranging from complete interfertility (*Hieracium echioides* [62]) via reduced fitness and fertility (*Arabidopsis thaliana* [46], *Chamerion angustifolium* [11], *Solanum chacoense* [63]) to almost complete failure of crosses (*Centaurea phrygia* [38], *Cyrtanthus breviflorus* [64], *Plantago media* [65]). In contrast, crosses between diploids and hexaploids consistently fail (*Arabidopsis thaliana* [46], *Aster amellus* [16]). However, there is much less known about reproductive success of heteroploid crosses in polyploid complexes encompassing more than one polyploid cytotype. Whereas diploids, tetraploids and hexaploids of the *Leucanthemum pluriflorum* clan [39], closely related diploid, tetraploid and octoploid species of *Castilleja* spp. and diploid to dodecaploid taxa belonging to the *Aster occidentalis* complex [66], [67] showed complete or nearly complete interfertility (but low germination of seeds resulting from heteroploid crosses in *Castilleja* [67]), diploid, tetraploid and hexaploid cytotypes of *Mimulus glabratus* failed to cross or produced infertile offspring [68]. However, for these systems the origin of polyploids is not fully resolved and may involve allopolyploidy. Thus, to our knowledge, *J. carniolica* is the first natural entirely autopolyploid system where reproductive interactions among three cytotypes have been comprehensively investigated by performing the full set of *in situ* reciprocal crosses.

Postmating isolation mechanisms in *J. carniolica* provide a strong barrier to gene flow between diploids and polyploids and might explain the rarity of intermediate cytotypes in nature despite a high number of contact zones [49]. As diploids and polyploids frequently co-occur in close proximity [48], [49] and have largely overlapping flowering periods (M. Sonnleitner et al., pers. obs.), additional mechanisms are expected to be at work that prevent fitness reduction due to the loss of gametes in infertile heteroploid crosses. Assortative mating by means of pollinator discrimination could reduce such losses, but generalistic behaviour of alpine pollinators [57] renders this effect unlikely. Alternatively, apomixis or autogamy frequently accompany polyploidisation and are considered to enforce lineage integrity [69]. In polyploid *J. carniolica*, however, we neither found evidence for apomixis (based on FCM profiles of seed analyses, i.e. the endosperm/embryo ploidy ratio, and amplified fragment length polymorphisms; M. Winkler et al., unpublished data) nor for a breakdown of self-incompatibility (Table 1). Instead, we observed a multilateral mentor effect [70], i.e. an increased selfing in the presence of heteroploid pollen, compared to SI-treatments (Table 1), especially in diploid-hexaploid crosses, where the majority of seeds originated from selfing rather than hybridisation. Similar mentor effects have also been documented in other diploid and polyploid taxa [71], [72], possibly counteracting fitness loss in the recipient of heteroploid pollen due to stigma clogging. Factors to remedy fitness loss in the pollen donor due to pollen loss remain, however, elusive.

Full interfertility of tetraploids and hexaploids suggests the absence of postmating isolation mechanisms between the polyploid cytotypes. Depending on vigour and fertility, pentaploids, which are restricted to areas of immediate contact (M. Sonnleitner & M. Winkler, pers. obs.), may determine the dynamics of such contact zones [73]. These include competition with parental cytotypes (pentaploid seedlings perform consistently better than tetraploid ones: Figure 1), mediating gene flow between the polyploid cytotypes, potentially affecting their ecological niches, or the establishment of a moving contact zone due to unidirectional backcrossing, which ultimately may expand the distribution of one and narrow the distribution of the other cytotype [74].

Our results might be biased because they were derived from a single sampling site only. However, the emerged pattern of cross-compatibility matches the co-occurrence pattern of cytotypes in the Eastern Alps [49] suggesting that the observed pattern of cross-compatibilities likely are valid for the entire distribution range.

### Dosage Effect

The observed pattern of cross-compatibility in heteroploid crosses of *J. carniolica*, evaluated by means of seed viability and seedling survival, can be explained by genome dosage effects. It matches the general expectation of a positive correlation between the magnitude of detrimental effects on seedling development and the deviation from the homoploid parental genome ratio of 2 m : 1p [46]. Thus, the almost complete failure of reciprocal diploid-hexaploid crosses reflects a 3-fold deviation (0.67 m : 1p, 6 m : 1p), low hybrid viability in diploid-tetraploid crosses corresponds to a 2-fold deviation (1 m : 1p, 4 m : 1p), and the lowest deviation (0.67-fold) in tetraploid-hexaploid crosses (1.33 m : 1p, 3 m : 1p) does not result in any fitness loss of hybrids. Similar patterns were observed in *Arabidopsis*, where crosses of diploids with tetraploids produced viable embryos, whereas crosses of diploids with hexaploids resulted in embryo abortion [46] (no crosses were, however, conducted between tetraploids and hexaploids). There is evidence for relaxed reproductive barriers between tetraploids and hexaploids (i.e. presence of pentaploids) as compared to diploids and polyploids in *Centaurea* [72] and *Knautia* [15]. These results are in line with the observation that deviations from the 2 m : 1p genome ratio in the endosperm can be tolerated to some extent [46].

An alternative explanation for different compatibilities among cytotypes may be sought in allelic differences. The diploid cytotype is genetically more distant from the polyploids than tetraploids and hexaploids are from each other (M. Winkler et al., unpublished data). Therefore, Bateson-Dobzhansky-Muller incompatibilities [75], pollen-pistil incompatibilities and/or impaired pollen tube growth [76], or nuclear-cytoplasmic interactions [77] may play a role as well.

In contrast to seed viability and seedling survival, seedling growth, estimated from time to first leaf and size of seedlings, was affected by the direction of crosses. This was particularly pronounced in crosses of diploids and tetraploids (Figure 1, Table 4). Triploids emerging from crosses of 2*x*4*x* grew faster and reached larger sizes than the average of parental treatments, whereas triploids emerging from 4*x*2*x* grew slower and remained smaller than the average of parental treatments. This pattern meets the expectations of complementary offspring phenotypes for reciprocal crosses in that paternal excess will result in large embryos [42], [46], [47], which may allow faster growth and larger size, whereas small embryos due to maternal excess grow more slowly and remain smaller. An alternative explanation for these differences are cytoplasmic effects resulting in hybrid trait values similar to those of the maternal parent [78]. This is consistent not only with trait values in offspring from diploid-tetraploid crosses, but would also explain the smaller seedlings in 4*x*6*x* crosses (Figure 1, Table 4) despite paternal excess.

### Unreduced Gametes

The progeny derived from experimental crosses among cytotypes of *J. carniolica* showed a remarkable diversity of ploidies as observed in heteroploid crosses of other species [32], [62], [72]. Apart from intermediate ploidy (hybrid offspring) and maternal ploidy (self-fertilised offspring), the offspring of heteroploid crosses also contained ploidies best explained by the involvement of unreduced gametes. Almost all unreduced gametes involved in successful heteroploid crosses originated from the diploid parent, mainly in the form of unreduced pollen. Unreduced gametes have also been reported from other polyploid systems (e.g., refs. [32], [72], [79]), but their evolutionary impact is difficult to predict because the frequency of unreduced gametes differs strongly among taxa [80], [81], among individuals and even within them [82], [83]. In *J. carniolica*, mating success of unreduced gametes appears to depend on the presence/absence of heteroploid pollen. For instance, a high proportion of seeds (16 out of 57) originated from 6*x*2*x* crosses were pentaploid and, thus, implied the involvement of unreduced diploid pollen, but no evidence for unreduced gametes was found in homoploid control treatments. Very few pentaploids were found in natural diploid-hexaploid populations (3 out of 1595 individuals; [49]), which may indicate low *in situ* formation and/or low survival rates (no seedlings were investigated by Sonnleitner et al. [49]).

## Conclusions

Although our conclusions are drawn from the analysis of plants originating from a single mixed-ploidy site, the degree of postzygotic reproductive isolation seems to vary strongly among cytotypes of *J. carniolica*. A likely underlying cause is the dosage effect in the endosperm [46], because cross-compatibility decreases with increasing deviation from the balanced ratio of maternal to paternal genome and trait values of early seedling stages are largely congruent with differences in seed size expected for reciprocal heteroploid crosses. Thus, the isolation between diploids and polyploids might be an immediate consequence of polyploidisation, although allelic differentiation and/or reinforcement in secondary contact zones [84] cannot be excluded. Irrespective of the underlying mechanisms, cross-compatibility patterns among cytotypes, together with ecological segregation and postglacial (re)colonisation history, will affect the distribution of cytotypes. This is evident from the frequent syntopic occurrence of the nearly fully incompatible diploids and hexaploids compared to the rare co-occurrence of the fully compatible tetraploids and hexaploids, where pentaploid hybrids will determine the evolutionary dynamics of contact zones. The dependence of heteroploid cross-compatibility on the parental ploidy described here for the autopolyploid complex of *J. carniolica* determines the long-term stability of contact zones and their roles in autopolyploid diversification and speciation.
